# The miRNAome of durum wheat: isolation and characterisation of conserved and novel microRNAs and their target genes

**DOI:** 10.1186/s12864-016-2838-4

**Published:** 2016-07-22

**Authors:** Domenico De Paola, Diana L. Zuluaga, Gabriella Sonnante

**Affiliations:** Institute of Biosciences and Bioresources, National Research Council, Via G. Amendola 165/A, 70126 Bari, Italy

**Keywords:** microRNAs, Durum wheat, Sequencing, Bioinformatics analysis, qPCR expression analysis, Target genes, RNA derived miRNAs

## Abstract

**Background:**

The allotetraploid durum wheat [*Triticum turgidum* subsp*. durum* (Desf.) Husn.] is a highly economically important species especially in the Mediterranean basin. However, its genomics, transcriptomics and in particular microRNAome are still largely unknown.

**Results:**

In the present work, two small RNA libraries from durum wheat Ciccio and Svevo cultivars were generated from different tissues at the late milk (Z77) developmental stage. A total of 167 conserved and 98 potential novel miRNAs were identified in the two libraries and interestingly, three novel miRNAs were found to be derived from ribosomal RNA. Putative target genes were predicted for conserved and novel miRNAs, the majority of which interact with nucleic acids, according to GO terms relative to molecular function. Quantitative qPCR analysis showed that several miRNAs identified were differentially expressed in the mature (Z77) developmental stage compared to young (Z14) tissues. Moreover, target gene expression analysis suggested that in roots, the putative genes encoding for the SQUAMOSA SPL2 and TGA1 proteins are regulated by ttu-miR156n, while MYB3 transcription factor by ttu-miR319f. Additionally, the Photosystem II P680 chlorophyll A apoprotein gene showed an expression level negatively correlated to that of ttu-novel-48 in leaves.

**Conclusion:**

Our results suggest that, in durum wheat, these genes may play important roles in root/leaf development and are subjected to miRNA regulation. The prediction of novel miRNAs putatively derived from ribosomal RNA opens new perspectives on the study of plant miRNAs.

**Electronic supplementary material:**

The online version of this article (doi:10.1186/s12864-016-2838-4) contains supplementary material, which is available to authorized users.

## Background

Durum wheat [*Triticum turgidum* subsp*. durum* (Desf.) Husn.] is the most widespread crop in the Mediterranean basin, since it is the base for the production of pasta [1] and other typical products of this area such as breads and cuscus. Durum wheat is an allotetraploid species (AABB) which derives from the cross between *T. urartu* (diploid with A genome) and an ancient relative of *Aegilops speltoides* Tausch*.* (diploid and donor of the B genome). In spite of the economic importance of durum wheat, there is still a lot to know about the genomics and transcriptomics of this species. In particular, information on the microRNAome of durum wheat is lacking. MiRNAs are small non-coding RNA molecules which downregulate gene expression by mRNA degradation or translational repression. Several conserved miRNAs regulate genes encoding transcription factors with important roles in a variety of processes in plants, including protein degradation, response to biotic and abiotic stresses, signal transduction, and developmental processes [2–7]. miRNAs are well studied in the reproductive parts of plants, where they regulate flowering time [8], seed [9, 10] and flower [11] development.

The key role of miRNAs in developmental processes has been widely demonstrated in model plants and crops including some species of the Poaceae family. In rice, miR156 regulates flowering time and temporal expression changes of numerous genes during leaf development [12], whereas miR397 and miR528 were detected to have an important role in rice seed development [13]. In barley, miR160 potentially targets the auxin response factors (ARF) 10 and 16, which have critical roles in plant root development [5, 14]. Additionally, barley miR171 targets a plant-specific scarecrow-like transcription factor 6 (SCL6) which regulates several plant developmental processes [15, 16]. In maize, it has been proved that miR172 downregulates glossy15 to promote vegetative phase change [17].

The widespread use of high throughput sequencing has promoted advancements in the genomics of plants, including those species with large, repetitive and polyploid genomes as wheat [18]. The recent availability of the hexaploid bread wheat (*T. aestivum* L.) draft genome has sped up the discovery of small RNAs in this species in the past few years [19–22]. Thus, so far, 119 mature miRNAs are annotated for *T. aestivum* in the miRNA repository miRBase (http://www.mirbase.org/, release 21). Several studies have uncovered development-associated miRNAs in bread wheat mainly in the seeds, thus being potentially involved in the regulation of grain-filling [23], and preferentially expressed in developing or germinating seeds [24].

Conversely, a limited number of studies have been carried out on miRNAs in tetraploid wheats. For instance, miRNA expression patterns in response to drought stress were assessed in leaf and root of wild emmer wheat [*T. turgidum* L. subsp. *dicoccoides* (Körn. ex Asch. et Graebn.) Thell. ], the ancestor of durum wheat, through a plant miRNA microarray platform and a homology-based approach [25]. Additionally, the same group performed an *in silico* identification of several miRNAs from root transcriptome of *T. turgidum* ssp. *durum* and *T. turgidum* ssp. *dicoccoides* [26]. In spite of this, the discovery of miRNAs in durum wheat is still largely unknown, with just one miRNA reported in miRBase release 21.

The aim of this study was to identify conserved and novel miRNAs in durum wheat, and evaluate their possible involvement in plant development. To this purpose, two small RNA libraries from the cultivars Ciccio and Svevo were generated from a pool of different tissues at the late milk stage [27]. Following high throughput sequencing, conserved and novel miRNAs and their target genes were identified. Some miRNAs were predicted to regulate genes involved in plant development or in important biochemical processes such as photosynthesis. Interestingly, we found three novel miRNAs derived from ribosomal RNA in durum wheat. In order to investigate the possible involvement of selected conserved and novel miRNAs and target genes in the development of leaves and roots, quantitative PCR (qPCR) was performed to highlight differences in expression profiles. This work provides useful information that may be employed in breeding programs aiming at improving durum wheat development.

## Methods

### Plant material and growth conditions

Seeds of two durum wheat cultivars, Ciccio and Svevo, were sterilized for 2 min in ethanol and then for 3 min in 2 % sodium hypochlorite. After 12 h in water aired with an oxygen air pump, seeds were transferred into 55 mm petri dishes with a wet filter paper and kept for 10 days at 4 °C.

Once the seed hypocotyl became half centimetre, plantlets were transferred into 10 cm diameter net pots containing a mix of 80 % agriperlite and 20 % expanded clay, and placed in a greenhouse at 18–20 °C under 12 h photo-period. Plants were grown in a hydroponic system containing 0.65mM MgSO_4_.7H_2_O, 0.75mM K_2_SO_4_, 0.1mM KH_2_PO_4_, 2mM Ca(NO_3_)_2_.4H_2_O, 0.05μM (NH_4_)_6_Mo_7_O_24_.4H_2_O, 10μM H_3_BO_3_, 1μM MnSO_4_, 1μM ZnSO_4_, 5μM CuSO_4_, 100μM Fe-EDTA and a pH = 6. Roots were submerged all the time in the solution and this was refreshed every two days.

At the Z14 stage of seedling growth [27], roots and leaves were collected from three individual plants from Ciccio and Svevo separately. At the late milk developmental stage (Z77), roots, leaves, flag leaves, and spikes were collected as above. All tissues were immediately frozen in liquid nitrogen and stored at −80 °C.

### Small RNA extraction and library preparation

Small RNAs were isolated from 100 mg of each tissue from each durum wheat plant at the stage Z77, using the mirPremier microRNA Isolation Kit (Sigma-Aldrich, St. Louis, MO, USA). RNA quality was verified by agarose gel according to manufacturer’s instructions. The small RNA fractions from the four tissues of the three plants were pooled for library preparation.

TruSeq Small RNA Sample Preparation (Illumina, San Diego, USA) protocol was used for the construction of two small RNA libraries from Ciccio and Svevo tissues, respectively. Libraries were sent for sequencing to the Institute of Applied Genomics (Udine, Italy).

### Data analysis and identification of conserved and novel miRNA

All reads were filtered and trimmed in order to discard low quality sequences and adapters. Unique small RNAs were counted eliminating redundancies and each count was normalized and expressed as transcript per million (TPM). In order to identify conserved miRNAs, unique reads were subjected to similarity search using all plant mature miRNAs from miRBase (release 21) as reference database. To this purpose, Blastn was used with parameters optimized for short sequences. Only perfect matching sRNA were extracted and sequences with at least 5 counts in one of the two libraries were considered for further analysis. Potential novel miRNAs were identified using a custom pipeline. The potential precursor sequences of novel durum wheat miRNAs were obtained by Blastn search against all the ESTs from the NCBI database. This similarity search was also extended to *T. aestivum* unigenes in the DFCI Gene Index (TAGI), version 12 (ftp://occams.dfci.harvard.edu/pub/bio/tgi/data/Triticum_aestivum/TAGI.release_12.zip). All sequences identified were subjected to UNAfold [28] to predict their secondary structure. To select conserved and novel miRNAs in durum wheat we used the criteria for plant miRNA annotation described by Meyers et al. [29].

### qPCR quantification of microRNAs

For the qPCR validation of selected miRNAs, Svevo was chosen since it is one of the most studied durum wheat varieties, and is well-known for its qualitative properties, e.g. high protein content and high yellow index [30].

Small RNAs were extracted using the mirPremier microRNA Isolation Kit (Sigma-Aldrich, USA) and miRNAs were detected using stem-loop RT-PCR method [31]. Small RNA quality was checked by electrophoresis through a 1 % agarose gel containing 1 % gel red (Biotium, Hayward, CA, USA). Quantitative qPCR analyses were performed using the Universal Probe Library (UPL) probe assay specific for mature miRNA expression, described by Varkonyi-Gasic and colleagues [31]. Each reaction was performed in a final volume of 10 μl containing 5 μl of Rotor-gene Probe PCR Master Mix 2x (Quiagen, Hilden, Germany), 0.5 μl of miRNA-specific forward primer (10 μM) and the universal reverse primer (10 μM) (Additional file 1), 0.1 μl of UPL, Probe #21 (Roche Diagnostics GmbH, Mannheim, Germany), 3.4 μl of nuclease-free water, and 0.5 µl of the respective retrotranscribed miRNA. Primers for gene expression analysis were designed based on sequences obtained from the library analysis (Additional file 1). PCR conditions were: 95 °C for 5 min, followed by 40 cycles of 95 °C for 5 s, 60 °C for 10 s and 72 °C for 1 s. Reactions were performed using a Rotor-Gene 6000 machine (Corbett Life Science, Sydney, Australia). Three technical replications of three different plants and a no-template control were included for each experiment. The housekeeping miRNA was chosen based on expression data using qPCR of eight different putative reference miRNAs (miR319, miR159, miR164, miR156, miR444, miR167, miR171 and miR160) previously described in wheat [32, 33]. Data were analysed using GeNorme and NormFinder software and miR159 showed to be the most stable miRNA in young and mature tissues of Svevo roots and leaves in our experimental conditions. Relative quantification of each single miRNA expression was analysed using the comparative C_T_ method as described by Livak and Schmittgen [34]. Differentially expressed miRNAs were identified as those showing > 2-fold change between the two developmental stages. After performing independent Student’s *t* test, fold changes with a *p*-value < 0.05 were considered statistically significant.

### Prediction of ttu-miRNA target genes

Novel and conserved durum wheat miRNAs were used as query in the online tool psRNATarget (http://plantgrn.noble.org/psRNATarget/) [35]. The analysis was performed using three different transcript libraries for target search: durum wheat ESTs from the NCBI database, *T. aestivum* unigenes from DFCI Gene Index (TAGI) version 12, and *Hordeum vulgare* unigenes from DFCI Gene Index (HVGI) version 12. Blast2GO [36] was used to obtain GO annotation of the wheat unigenes based on BLASTx hits against the NCBI nr database. GO functional classification was obtained executing mapping and annotation steps in BlastGO with default parameters. Two graphs representing GO terms categories (molecular function and biological processes) were determined for all durum wheat miRNA targets.

### Expression analysis of target genes

The expression of miRNA putative target genes was analysed using the SYBR Green dye and the Rotor-Gene 6000 machine (Corbett Research, Australia). Primers for gene expression analysis are found in Additional file 1. Total RNA was isolated using the RNeasy Mini Kit (Qiagen, Valencia, CA) and reverse transcribed into cDNA, using the Script cDNA Synthesis Kit (Bio-Rad Laboratories, Richmond, CA) according to the manufacturer’s instructions. Before expression analysis, the same primers were used for sequencing of the target gene fragments including the miRNA binding site. Real-time qPCR experiments were carried out using 20 ng of cDNA and the iQ SYBR Green Supermix (Bio-Rad Laboratories, Richmond, CA) following the manufacturer’s protocol. PCR conditions were: 95 °C for 3 min, followed by 40 cycles of 95 °C for 10 s and 58 °C for 30 s. The reference gene was chosen by testing three putative reference genes, i.e. Actin, Glyceraldehyde-3-Phosphate Dehydrogenase (GAPDH) and cell division control protein, AAA-superfamily of ATPases (CDC), through a qPCR expression analysis in leaves and roots of Svevo young and mature tissues. Data were analysed using GeNorme and NormFinder software suggesting CDC as the most stable gene in our experimental conditions. Therefore, using CDC as the reference gene, relative quantification of each single gene was performed using the comparative C_T_ method as described by Livak and Schmittgen [34]. Differentially expressed genes were identified as those showing > 2-fold change between the two developmental stages. After performing independent Student’s *t* test, fold changes with a *p*-value < 0.05 were considered statistically significant.

## Results

### Sequencing data analysis and identification of durum wheat conserved and novel miRNAs

In order to determine the expression of the microRNAome in durum wheat, two small RNA libraries were generated and sequenced from a mix of leaves, flag leaves, roots and spikes of Ciccio (SRA-NCBI accession SRR3144594) and Svevo (SRA-NCBI accession SRR3147015) plants, separately, at the developmental stage of late milk (Z77). Conserved and novel miRNAs and their target genes were identified using bioinformatics tools. After removing the low quality reads and adaptors, a total of 15,363,057 and 15,727,000 reads were collected from Ciccio and Svevo, respectively. The size distribution of the total clean reads in the two libraries (Additional file 2) shows a canonical trend, with the 21–24 nucleotide long sRNAs representing the majority of the total reads. The 24 nucleotide peak was the most abundant one, as observed in most plant species. Reads of the two libraries were searched against ncRNAs in Rfam (https://www.sanger.ac.uk/) to filter out the snoRNAs, snRNAs, tRNAs, and rRNAs before annotation with conserved plant miRNAs. As a result, 1,243,567 and 1,919,627 reads were discarded from the library of Ciccio and Svevo, respectively.

In order to identify conserved miRNAs in durum wheat, all the unique reads longer than 18 or shorter than 31 nucleotides were mapped to known plant miRNAs in miRBase release 21. A total of 167 known miRNAs were found in the two libraries, 166 in Ciccio and 162 in Svevo. Out of the conserved miRNAs identified, 135 belonged to 39 conserved miRNA families, whereas 32 were unassigned (Additional file 3).

**Fig. 1:**
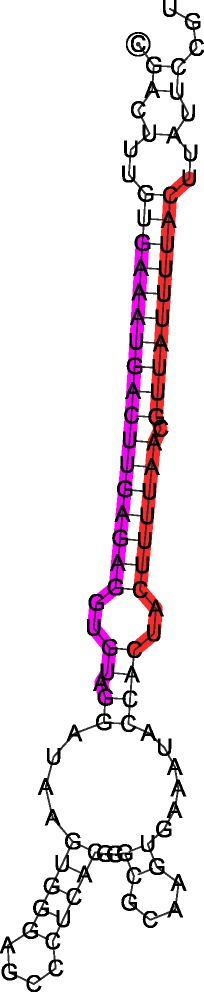
Pre-miRNA secondary structure of ttu-novel-48 (5′ arm) and ttu-novel-42 (3′ arm). 5′-end is marked within a circle

All the remaining reads, which were not annotated with miRBase, were used to identify potential new miRNAs using bioinformatics tools. Based on the analysis of the predicted secondary structure of durum wheat sequences, 98 potential miRNAs were considered as novel miRNA candidates (Additional file 4). This analysis revealed three non-canonical putative miRNAs (i.e. ttu-novel-42, ttu-novel-48, and ttu-novel-53). Similarity search of their precursor sequences matched to ribosomal RNA, which has never been reported as a source of plant miRNAs before. The analysis of the secondary structures and their predicted thermodynamic stability meet all criteria for *in silico* miRNA prediction [29]. Moreover, these novel miRNAs were detected in independent NGS libraries, both the ones used in this study, and other durum wheat sRNA proprietary libraries (data not shown). Two non-canonical miRNAs (i.e. ttu-novel-42 and ttu-novel-48) were mapped to the two opposite arms of the same sequence (Fig. 1). The precursor sequences of the novel miRNAs ttu-novel-53 (AJ613637.1) and ttu-novel-42/ttu-novel-48 (FL578040.1) were mapped to 18S rRNA and 28S rRNA, respectively. In order to verify whether other miRNAs present in miRBase could derive from rRNA, all plant miRNA precursors were blasted against the nucleotide database of Viridiplantae in NCBI. At least one miRNA, peu-MIR2916 from *Populus euphratica* matched with 18S RNA, showing 96 % identity with *Idesia polycarpa* gene for 18S ribosomal RNA, as the best hit.

### Expression analysis of selected microRNAs

Several of the conserved miRNAs found were reported to regulate development in a range of plant species. In order to highlight a possible involvement of these miRNAs in durum wheat cv. Svevo leaf and root development, we selected 15 of those for the analysis of relative expression by quantitative PCR (qPCR) at an early stage (Z14) and an advanced late milk developmental stage (Z77) [27]. Additionally, five novel miRNAs were selected based on their predicted target or according to their origin from rRNA molecules. This set of novel miRNAs was analysed from roots and leaves of Svevo plants in the two different stages.

In order to choose a suitable housekeeping miRNA, based on the literature [32, 33], eight miRNAs were selected and evaluated as putative housekeeping genes in our experiment: ttu-miR319, ttu-miR159, ttu-miR164, ttu-miR156, ttu-miR444, ttu-miR167, ttu-miR171, and ttu-miR160. The qPCR expression profiles of these miRNAs were analysed for their stability, and ttu-miR159 proved to be the most stable in our experimental conditions. Therefore, ttu-miR159 was chosen from this group as the housekeeping miRNA for our analyses.

**Fig. 2:**
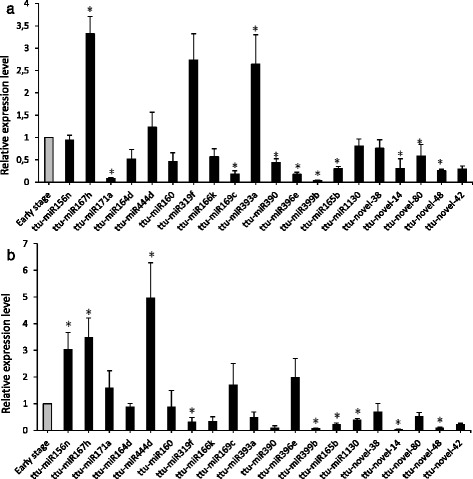
Relative expression analysis of selected miRNAs and target genes. Conserved and novel *T. turgidum* miRNAs in leaves (**a**) and roots (**b**) at the early stage (*grey bar*, arbitrarily set to 1) and at the mature stage (*black bars*). Expression levels are given as fold change of mature stage versus early stage. Data are means ± SE. *P* values between three early stage replicates and three mature stage replicates for each miRNA were calculated using unpaired, 2-tailed student’s *t* test

Results of miRNA expression analysis show that six conserved miRNAs (ttu-miR171a, ttu-miR169c, ttu-miR390, ttu-miR396e, ttu-miR399b, ttu-miR165b) were significantly downregulated in leaves at the mature stage compared to the leaves at the early stage, with fold changes ranging from 0.038 (ttu-miR399b) to 0.445 (ttu-miR390). Additionally, ttu-miR167h and ttu-miR393a were found to be significantly upregulated in leaves at the adult stage compared to the early stage, with fold changes of 3.325 and 2.639, respectively (Fig. 2a). For ttu-miR319f, although a higher expression level was observed in the Z77 leaves, this value was not significant (*p* = 0.334). On the other hand, in the roots, five conserved miRNAs (ttu-miR319f, ttu-miR390, ttu-miR399b, ttu-miR165b, ttu-miR1130) were found to be significantly downregulated at the Z77 stage compared to the Z14 stage, with fold changes ranging from 0.072 (ttu-miR399b) to 0.394 (ttu-miR1130). Ttu-miR166k was also strongly downregulated in roots (fold change 0.345), but with a p-value of 0.08 (not significant). In contrast, ttu-miR156n, ttu-miR167h and ttu-miR444d were found significantly up regulated in elder roots, with fold changes of 3.031, 3.482, and 4.982, respectively (Fig. 2b).

The analysis carried out for selected novel miRNAs confirmed the expression in durum wheat plants of the two putative rRNA-derived miRNAs previously identified, ttu-novel-42 and ttu-novel-48. In details, quantitative assay revealed that in leaves, two novel miRNAs, ttu-novel-14, ttu-novel-48 were significantly downregulated at the stage Z77, compared to the early stage Z14, with fold changes of 0.308 and 0.256, respectively (Fig. 2). In roots, ttu-novel-14 and ttu-novel-48 were strongly downregulated (fold change 0.041 and 0.100, respectively). Ttu-novel-42 showed a downregulation with a fold change of 0.294 and 0.221 in leaves and roots, respectively, but these values were not significant. For ttu-novel-38 and ttu-novel-80 the expression levels observed at the two developmental stages were comparable for both the tissues analysed.

### Prediction of miRNA specific target genes

In order to predict ttu-miRNA target genes, durum wheat ESTs, bread wheat unigenes and barley unigenes were used as distinct datasets, and subsequently results were analysed separately. Sequences carrying a region of high complementarity with the ttu-miRNAs were identified in the three databases, which were analysed with different priority starting from *T. turgidum* followed by *T. aestivum* and *H. vulgare*. A number of 6,889 target genes were predicted for all durum wheat miRNAs identified in this work, for a total of 3,516 and 3,373 sequences, matching conserved and novel durum wheat miRNAs, respectively (Additional file 5).

**Fig. 3:**
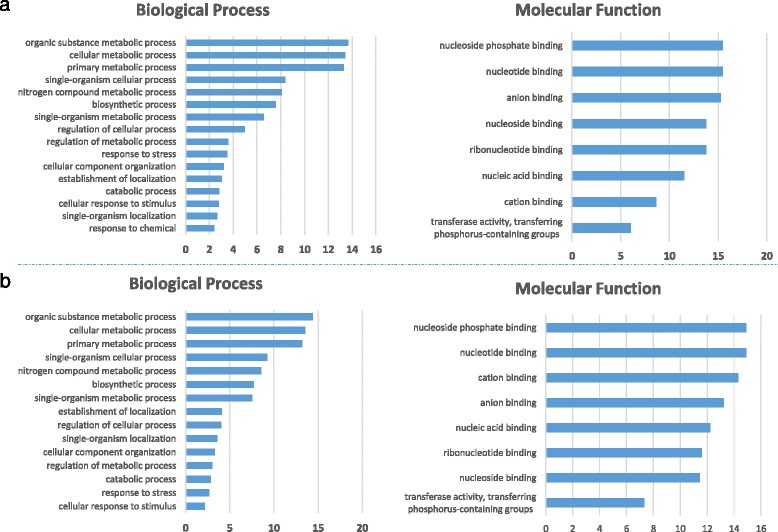
Bar charts representing Biological Processes and Molecular function categorization according to GO terms associated to durum wheat miRNA targets; bars represent the percentage of targets enriched for a specific GO term relative to the total number in the category. **a** GO annotation for conserved miRNA targets; **b** GO annotation for novel miRNA targets

MiRNA target genes predicted using wheat DFCI gene index were subjected to *in silico* functional analysis using Gene Ontology (GO) annotation. The assignment of GO terms highlighted different biological processes and molecular functions for novel and conserved miRNA targets. According to terms description relative to molecular function, the majority of the targets interact with nucleic acids. In fact, for both novel and conserved durum wheat miRNA target genes, nucleotide binding or nucleoside phosphate binding were major classes with approximately 15 % of total sequences. With regard to the other GO terms, there is a certain overlap of function in the comparison between novel and conserved miRNA targets, with some significant differences such as cation binding including 8.7 and 14.3 % targets for conserved and novel miRNAs, respectively (Fig. 3). With regard to the biological processes, the majority of the targets were annotated to terms related to metabolic processes. GO annotations partially overlap for novel and conserved miRNA targets, with the exception of the class “response to chemical”, which is missing in novel miRNA targets (Fig. 3b).

Some of the target genes identified *in silico* were possibly regulated by miRNAs showing a differential expression in leaves and/or roots between the two developmental stages. A number of these genes were chosen for further analysis. For instance, our target gene computational approach, identified that ttu-miR169c may regulate the homologue to HAP2 subunit (TC510142) of HAP complex. Another miRNA, ttu-miR156n, was predicted to regulate durum wheat genes encoding the SQUAMOSA promoter-binding-like proteins (SPL) SPL2, SPL16, SPL3, and SPL11, and a protein similar to the SPL teosinte glume architecture 1 (TGA1) of *Zea mays* (TC453361). Additionally, genes encoding for a homologue to the MIKC-type MADS-box transcription factor WM30 (TC424265), a flowering promoting factor-like 1 (CD880846) and the MYB3 (TC368630) transcription factor (TF) were predicted to be the target genes of ttu-miR444d, ttu-miR1130 and ttu-miR319f, respectively. Two genes encoding for the TIRA1 TIR1-like auxin receptor (TC422531) and a protein similar to the Auxin Signaling F-box 2 (ASF2) of Arabidopsis (TC257470) were identified as target genes of miR393a. Finally, three genes encoding for a Cytochrome P450 like-TBP (TC400716), the *Oryza sativa* early proembryo mRNA, similar to Rpe1 gene (AF45498.1) and a Photosystem II P680 chlorophyll A apoprotein (TC368631) were identified to be putative target genes of the ttu-novel-48.

### Expression of predicted target genes

Eleven predicted target genes of seven (six conserved and one novel) miRNAs differentially expressed between the two developmental stages and possibly involved in plant development, were considered for expression using qPCR analysis. Prior to expression analyses, the target gene fragments used for qPCR experiments were sequenced to confirm the miRNA binding site and the sequence of the predicted target genes.

The expression level of each gene was determined in roots and leaves of the late milk stage relative to the young stage. In general, more interesting results were observed in roots compared to leaves.

**Fig. 4:**
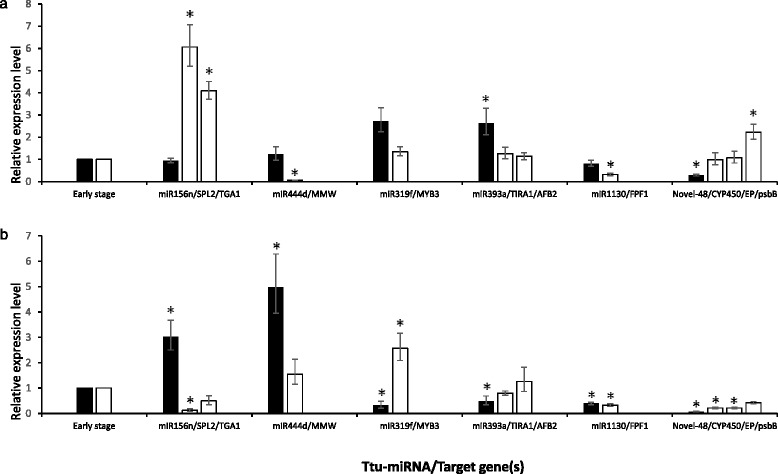
Several miRNAs differentially expressed and their target genes (white bars) in leaves (**a**) and roots (**b**) at the mature stage compared to the early stage (bars arbitrarily set to 1). Target genes are indicated by abbreviations: TGA1, teosinte glume architecture 1; AFB2, AUXIN SIGNALING F-BOX 2 protein; FPF1, Flowering promoting factor-like 1; MMW, MIKC-type MADS-box transcription factor WM30; TIRA1, TIR1-like auxin receptor; CYP450, Cytochrome P450 like_TBP; EP, early proembryo; psbB, Photosystem II P680 chlorophyll A apoprotein; SPL2, squamosa promoter-binding-like protein 2; MYB3, transcription factor Myb3. Expression levels are given as fold change of mature stage versus early stage. Data are means ± SE. *P* values between three early stage replicates and three mature stage replicates for each gene were calculated using unpaired, 2-tailed student’s *t* test

The putative target gene SQUAMOSA *SPL2* (CK196549) showed an evident strong downregulation (fold change = 0.122, *p*-value = 0.023) in roots of the adult plants compared to young roots, while ttu-miR156n was upregulated in the same tissue (Fig. 4), evidencing a possible correlation between the expression of the miRNA and this target gene. The other putative target of ttu-miR156n, *TGA1* (teosinte glume architecture 1) showed a non-significant variation in the expression level between the two developmental stages. On the other hand, both genes were highly upregulated in Z77 leaves compared to Z14 leaves, even though ttu-miR156n was not differentially expressed in this tissue in our conditions (Fig. 4).

The relative expression level of *MYB3* (TC368630), the putative target gene of ttu-miR319f, showed about two-fold increase (*p* = 0.035) in Z77 roots compared to the Z14 stage. This value was inversely correlated to the expression of ttu-miR319f, which showed an approximately two-fold decrease in the late milk stage. In the leaves, *MYB3* did not vary significantly and ttu-miR319f showed an expression level, whose variation was not significant.

Although MIKC-type MADS-box *WM30* (*MMW*) TF was highly downregulated in mature leaves, its expression did not appear to be correlated to that of the miRNA expected to regulate it, ttu-miR444d. In the roots, *MMW* did not show a significant variation in gene expression, while ttu-miR444d was upregulated.

*TIRA1*, TIR1-like auxin receptor, and the AUXIN SIGNALING F-BOX 2 (AFB2) protein are putative targets of ttu-miR393a. Both in leaves and roots, the two genes were not differentially expressed at the late milk stage compared to the early developmental phase, even though a significantly upregulation of ttu-miR393a was observed in the leaves.

The flowering promoting factor-like 1 (CD880846) was predicted to be the putative target of ttu-miR1130. However, the two expression analyses did not show any correlation (Fig. 4).

The three genes identified as putative targets of the ttu-novel-48 were also analysed by qPCR. In leaves, the Photosystem II P680 chlorophyll A apoprotein (psbB) was significantly upregulated in adult leaves (fold change 2.227, *p* = 0.047), and its miRNA was significantly downregulated, showing a correlation between the miRNA and its putative target. The other two predicted targets of ttu-novel-48, Cytochrome P450 like-TBP and early proembryo did not show differential expression in leaves. In roots, the three genes, as well as the ttu-novel-48 were downregulated.

## Discussion

MicroRNAs have proved to regulate a wide range of physiological processes in plants, including development, transition from one physiological phase to another, flowering time, etc. [3]. In durum wheat, little is known on the miRNA presence, abundance, and involvement in plant development.

Using massive sequencing of sRNA libraries of the two different durum wheat cultivars, Ciccio and Svevo, we obtained over 31 million filtered sequences, which allowed us to retrieve 167 conserved and 98 potential novel miRNAs. In order to understand whether some of the identified miRNAs could play a role in durum wheat plant development, especially in leaves and roots, the expression level of selected miRNAs was evaluated in a young stage (Z14) and in an adult stage (Z77). Most of the conserved miRNAs identified showed a differential expression between the early and the mature stage, and therefore they may play an important role in durum wheat development. Based on GO annotation of target genes of durum wheat miRNAs, various biological processes and molecular functions were predicted. As in most plant species, a large number of durum wheat miRNAs were expected to target transcripts annotated as nucleic acid binding proteins, several of which were homologs of known transcription factors controlling gene expression involved in plant development, morphology, and flowering time [37].

In plants, gene regulation accomplished by miRNAs can occur via target mRNA degradation and/or translational repression. The first mechanism usually arises when a strong sequence complementarity exists between the miRNA and its target, while imperfect pairing causes translational repression without mRNA cleavage [38]. Due to miRNA regulation, a negative correlation is observed between the expression level of a miRNA and its corresponding target. For this reason, qPCR experiments measuring the expression level of miRNAs and their targets are a valuable method to validate putative target genes [39–41]. In this work, we observed a negative correlation between the expression of some miRNAs and their putative target genes, especially in three cases: ttu-miR156n and its target SPL2, ttu-miR319f and the gene MYB3 in the roots, ttu-novel-48 and its putative target psbB in the leaves.

SQUAMOSA SPL genes encode plant-specific TFs involved in the transition from juvenile to adult phase and from vegetative to reproductive phase [42, 43]. Several SPL genes (i.e. AtSPL9) are post-transcriptionally regulated by miR156 [44]. Investigations on miR156/SPLs relationship published so far, have focused almost exclusively on their roles in plant aerial organs. However, recently, the important role of miR156/SPLs in *Arabidopsis* lateral root development has been unravelled [45]. These miR156/SPL feedback interactions throughout the development of plant aerial organs and roots may be conserved in other species [44]. Therefore, the upregulation of ttu-miR156n in the roots at the mature development stage and the downregulation of its putative target genes (SPL2, TGA1) in the same tissue, suggest a role of miR156/SPLs and miR156/TGA1 in root development of durum wheat, even though the downregulation of TGA1 (fold change 0.493) was not statistically significant. In *Arabidopsis* leaves, miR156 expression levels are high in the juvenile phase and decrease during shoot maturation [46]. It has to be noticed that in our durum wheat adult leaves, both *SPL2* and *TGA1* are strongly and significantly upregulated, however, ttu-miR156n is similar to the control. The higher expression of the *SPL* genes at the mature stage could be explained because of the role that *SPL* genes play in the control of lateral organs development in association with shoot maturation in the reproductive phase, as it has been demonstrated for SPL2 in *Arabidopsis* [47]. Further analysis should be carried out in order to understand the mechanisms of regulation of *SPL* genes in adult leaves of durum wheat.

Our findings suggest that ttu-miR319f might regulate the expression of *MYB3* in the roots of Svevo plants. Homology analysis showed that there is a high similarity between wheat *MYB3* and *Arabidopsis AtMYB59* [48]*.* It has been demonstrated that *AtMYB59* expression increases in response to phytohormones, including jasmonic acid and ethylene [49], which inhibit developmental processes such as cell growth. Therefore, the upregulation of ttu-miR319 at the immature stage and consequently the downregulation of its target gene MYB3 may be a consequence of the higher cell growth, cell division and DNA synthesis that characterizes an early developmental stage.

For ttu-novel-48 miRNA, we predicted *in silico* three putative target genes encoding for the Cytochrome P450 like_TBP, a protein similar to Rpe1 (AF45498.1), and the Photosystem II P680 chlorophyll A apoprotein (psbB gene). We tested all these potential targets in qPCR and evaluated their expression level compared to that of ttu-novel-48 miRNA. The significant upregulation of the psbB gene in the mature leaves suggests that the activity of the Photosystem II P680 chlorophyll A apoprotein is higher in the mature leaves of durum wheat, compared to young leaves. The lower expression of psbB in the leaves of the early stage could be a possible result of a higher target degradation by the ttu-novel-48 miRNA in the young leaves. As many processes of the plant, photosynthesis is targeted by a high number of miRNAs, as it was demonstrated in rice [50]. In monocots, there is a polarization in cell division and expansion being the oldest cells of the leaves photosynthetically more developed than the younger ones [51]. This phenomenon might explain why in durum wheat mature leaves, a higher expression of the gene was observed. Our data suggest that a possible regulatory mechanism of psbB gene dependent on ttu-novel-48 might occur in durum wheat leaves, even though deeper analyses need to be conducted in order to confirm that psbB is the target of this durum wheat novel miRNA. Ribosomal RNA (rRNA) is extremely abundant in the eukaryotic cells and constitutes about 80 % of the total cytoplasmatic RNA [52]. Small rDNA-derived RNAs (srRNAs) have been identified in mouse and human and they are involved in the regulation of metabolism and other biological processes [53, 54]. High-throughput sequencing of small RNA transcriptome reveals the existence of many different RNA fragments derived from small RNA. In many cases, these sequences are discarded as degradation products. Nevertheless, experimental evidence suggests that RNA fragments derived from small non coding RNAs such as nucleolar RNA (snoRNA) and transfer RNA (tRNA) are not just the result of RNA turnover but rather functional molecules [55]. To our knowledge, this is the first report on miRNAs deriving from rRNA in plants.

The expression level of the putative target genes of miR444d (*MMW*) and miR393a (*TIRA1* and *AFB2*) was evaluated since a differential expression of these miRNAs was observed in roots (miR444d) and both leaves and roots (miR393a). However, the expression analysis of their target genes did not show a correlation with the miRNA expression. MiR444 is specific to monocots and it targets four MIKC-type MADS-box genes in rice (*OsMADS23*, *OsMADS27a*, *OsMADS27b*, and *OsMADS57*), homologous to *Arabidopsis* ANR1 [56–58]. In *Arabidopsis*, it has been shown that ANR1 is a major component in the NO_3_^−^-signalling pathway that triggers lateral root growth. In this work, ttu-miR444 was predicted to regulate genes encoding the MIKC-type MADS-box TFs (*WM30*, *WM32B* and *WM32A*), homologues of the *T. aestivum* MIKC-type MADS-box TFs. Ttu-miR444d clearly shows to be highly upregulated in roots of durum wheat in an advanced developmental stage, although no inverse correlation was observed with its putative target *WM30*. Probably, this miRNA targets another member of the MIKC-type MADS-box TFs, or a post-transcriptional regulation might occur. MiR444 has been shown to play multiple roles in the rice NO_3_^−^-signalling pathway involving root development, nitrate accumulation and Pi starvation responses [59]. Overexpression of miR444 resulted in a reduction of rice lateral root elongation, but in a promotion of rice primary and adventitious root growth, in a nitrate-dependent manner. Therefore, it may be that in durum wheat, in the first developmental stages, plants are still more active in developing primary and adventitious roots than lateral roots. MiR393 regulates the activity of TIR/AFB2 (TRANSPORT INHIBITOR RESPONSE1/AUXIN SIGNALING F-BOX PROTEIN2) Auxin Receptor (TAAR) family F-box proteins in *Arabidopsis* leaves. The perception and signalling of auxin, a hormone implicated in plant development, depends on these receptors [60]. In our work, we detected a TIRA1 and an AFB2 gene from durum wheat, by blasting our miRNA data against *T. aestivum* and *H. vulgare* transcripts, respectively. Ttu-miR393a showed to be more expressed in the leaves at an advanced developmental stage compared to the early stage, so that TAAR genes are expected to be more active when durum wheat leaves are still in an immature stage. However, in our analysis, no variation was detected in the expression levels of the predicted target genes. This could be due to the presence of other isoforms of TAAR genes, which were not considered, or to the fact that notwithstanding perfect complementarity with mRNA, in some cases miRNA can inhibit translation irrespective of mRNA cleavage [61]. In the roots, we detected a very low miR393 expression. This fact is in agreement with what was observed in other species, e.g. artichoke where this miRNA was not detected [62].

In conclusion, some of the miRNAs identified in durum wheat are differentially expressed in leaves and roots of the two developmental stages analyzed, indicating a regulation in the plant development. For three miRNAs, a negative correlation with their target was observed. In other cases, a post-transcriptional regulation could occur. Interestingly, three novel miRNAs were predicted to derive from rRNA, and this could open new perspectives in the study of plant miRNA.

## Conclusions

The analysis of the durum wheat miRNAome from Ciccio and Svevo tissues at the late milk stage allowed to identify 167 conserved and 98 potential novel miRNAs. Several miRNAs identified were differentially expressed in the mature (Z77) developmental stage compared to young (Z14) tissues. Our results suggest that in roots, the putative genes encoding for the SQUAMOSA SPL2 and TGA1 proteins are regulated by ttu-miR156n, while MYB3 transcription factor by ttu-miR319f. Additionally, the Photosystem II P680 chlorophyll A apoprotein gene showed an expression level negatively correlated to that of ttu-novel-48 in leaves. These genes might play important roles in root/leaf development in durum wheat. The prediction of novel miRNAs putatively derived from ribosomal RNA opens new perspectives on the study of plant miRNAs.

## Abbreviations

ARF, auxin response; ASF2, auxin signaling F-box 2; CDC, cell division control protein; EDTA, ethylenediaminetetraacetic acid; GAPDH, glyceraldehyde-3-phosphate dehydrogenase; GO, gene ontology; HVGI, *Hordeum vulgare* unigenes from DFCI Gene Index; miRNA, MicroRNA; psbB, photosystem II P680 chlorophyll A apoprotein; qPCR, quantitative PCR; rRNA, ribosomal RNA; SCL6, scarecrow-like transcription factor 6; SPL, SQUAMOSA promoter-binding-like proteins; TAGI, *Triticum aestivum* unigenes from DFCI Gene Index; TF, transcription factor; TGA1, teosinte glume architecture 1; TIR/AFB2, transport inhibitor response/auxin signalling F-box protein2.

## Additional files

Additional file 1: Table S1. List of primers used for Q-RT-PCR assay for durum wheat miRNAs and their putative target genes. (XLSX 14 kb) Additional file 2: Figure S1. Length distribution of small RNA sequences in two libraries obtained from durum wheat Ciccio and Svevo cultivars. (DOCX 15 kb) Additional file 3: Table S2. Conserved miRNAs identified in durum wheat Ciccio and Svevo cultivars by Illumina sequencing. MiRNAs were annotated according to miRBase release 21. (XLSX 16 kb) Additional file 4: Table S3. Novel miRNAs from durum wheat identified in durum wheat Ciccio and Svevo cultivars by Illumina sequencing. MiRNAs were predicted *in silico* using similarity search and secondary structure analyses of putative pre-miRNAs sequences. (XLSX 51 kb) Additional file 5: Table S4. Putative target genes of durum wheat miRNAs identified *in silico.* The analysis was performed on three datasets: durum wheat ESTs from the NCBI database, *T. aestivum* unigenes from DFCI Gene Index (TAGI) version 12, and *Hordeum vulgare* unigenes from DFCI Gene Index (HVGI) version 12. (XLSX 648 kb)
