# Supplementary material for: The miRNAome of durum wheat: isolation and characterisation of conserved and novel microRNAs and their target genes
Source: BMC Genomics. 2016 Jul 22;17:505. doi: 10.1186/s12864-016-2838-4 (PMC4957382; doi:10.1186/s12864-016-2838-4)
Supplement: Additional file 2: Figure S1. — Length distribution of small RNA sequences in two libraries obtained from durum wheat Ciccio and Svevo cultivars. (DOCX 15 kb) [file 12864_2016_2838_MOESM2_ESM.docx]

**Figure Suppl. 1.** Read size distribution
